# Findings from an Organizational Context Survey to Inform the Implementation of a Collaborative Care Study for Co-occurring Disorders

**DOI:** 10.1007/s11414-023-09851-6

**Published:** 2023-08-03

**Authors:** Sandra K. Evans, Alex Dopp, Lisa S. Meredith, Allison J. Ober, Karen C. Osilla, Miriam Komaromy, Katherine E. Watkins

**Affiliations:** RAND Corporation, Santa Monica, CA, USA; RAND Corporation, Santa Monica, CA, USA; RAND Corporation, Santa Monica, CA, USA; RAND Corporation, Santa Monica, CA, USA; Department of Psychiatry and Behavioral Sciences, Stanford University School of Medicine, Palo Alto, CA, USA; Boston Medical Center, Grayken Center for Addiction, One Boston Medical Center Place, Boston, MA, USA; RAND Corporation, Santa Monica, CA, USA

## Abstract

Primary care is an opportune setting to deliver treatments for co-occurring substance use and mental health disorders; however, treatment delivery can be challenging due multi-level implementation barriers. Documenting organizational context can provide insight into implementation barriers and the adaptation of new processes into usual care workflows. This study surveyed primary care and behavioral health staff from 13 clinics implementing a collaborative care intervention for opioid use disorders co-occurring with PTSD and/or depression as part of a multisite randomized controlled trial. A total of 323 completed an online survey for a 60% response rate. The Consolidated Framework for Implementation Research guided this assessment of multi-level factors that influence implementation. Most areas for improvement focused on inner setting (organizational level) constructs whereas individual-level constructs tended to be strengths. This work addresses a research gap regarding how organizational analyses can be used prior to implementation and provides practical implications for researchers and clinic leaders.

## Introduction

Co-occurring mental health and substance use disorders are prevalent, have serious consequences, and remain undertreated—especially with evidence-based practices (EBP).^1–4^ Many individuals with opioid use disorder (OUD) have co-occurring disorders such as post-traumatic stress disorder (PTSD) and/or major depressive disorder (MDD).^5–7^ Most individuals with OUD have experienced trauma and close to two-thirds go on to experience co-occurring PTSD.^6–8^ Approximately half of those with OUD also have lifetime major depression^9^ and 20% have a 12-month diagnosis of a mood disorder.^10^ Undiagnosed and untreated co-occurring disorders may interfere with treatment of OUD and with return to baseline functioning.

Primary care is an opportune setting to deliver treatments for co-occurring substance and mental health disorders because individuals with substance use disorders, including OUD, tend to access primary care more frequently than specialty substance use settings.^11,12^ The Collaboration Leading to Addiction Treatment and Recovery from Other Stresses (CLARO) initiative is one of the first studies to evaluate a collaborative care model for co-occurring disorders in primary care settings.^13,14^ The multisite CLARO randomized controlled trial (RCT) assesses the effectiveness of a collaborative care intervention for OUD co-occurring with PTSD and/or depression, implemented in low-resource settings.^11,15^

Various factors within any organizational setting may influence the implementation of interventions to treat those with co-occurring disorders. In primary care, unique challenges may surface in part because both substance use and mental health disorders tend to be underidentified and undertreated in primary care settings. For example, patient characteristics, such as having multiple disorders that may interact, might pose a challenge to primary care providers or the organization (health system) itself may not be adequately equipped to treat individuals with co-occurring disorders. Broadly, factors like the organizational culture of a clinic may impact the extent to which new practices are adopted by providers. Similarly, an intervention may show positive outcomes however lack of leadership support may complicate the extent to which practices are sustained even if financial resources are stable. For example, previous research on co-occurring substance use disorder treatment has documented contextual barriers including the importance of administrative leadership; challenges related to staff turnover, training and supervision; and financial considerations like potential reductions in billing or process changes to financial services.^16^ These potential factors, while not necessarily exclusive to co-occurring disorder interventions, point to the value of understanding contextual factors in relation to interventions for co-occurring disorder.

Understanding the range of contextual factors, or determinants, of sites adopting an EBP can be critical to the success of its implementation.^17^ The first 2 years of CLARO (2020–2021) focused on implementing the collaborative care model in 13 clinics operating within three distinct healthcare systems in a western US. Understanding the context of these clinics early in the intervention implementation process was considered an important component of implementing the CLARO RCT. In particular, it seemed probable that organizational factors of the participating sites might impact the way collaborative care was implemented at each site, as well as its potential sustainability.

For these reasons, it is vital to understand the landscape of each organization prior to implementing an intervention and treat it as part of the implementation planning process. Identifying potential barriers and facilitators to implementation may reveal areas that are in need of additional support or that should be tracked over time.^18,19^ This study contributes to the developing research area that links pre-implementation research to implementation plans.^17^ In a study aimed at testing psychometric Consolidated Framework for Implementation Research (CFIR) measures, Fernandez et al.^20^ argue that inner setting constructs—those that focus on organizational context specifically—can be used to inform or enhance implementation approaches; for example, shining a light on deficient constructs can lead to making or measuring changes to those areas over time. Weir et al.^21^ took this approach further by identifying implementation barriers and then developing strategies based on the CFIR to help facilitate the implementation of a cluster randomized trial in hospitals.

The current study describes results of a survey administered in three healthcare systems during the pre-implementation phase designed to assess contextual factors that may influence implementation of the CLARO intervention. Guided by the well-established CFIR^19,22,23^ comprising 37 characteristics across five domains believed to influence implementation of EBPs, this research examines the question, how do perceptions of contextual factors vary among the three systems, and across types of staff? The discussion section also addresses how the research team used these results to inform the RCT.

## Methods

### Overview of study design

The purpose of the survey was to develop a pre-implementation understanding of organizational context of clinics participating in the CLARO RCT. The target population for the survey was clinic staff in the primary care clinics, including clinicians. The intent was to survey as many staff members from each healthcare system as possible, and to gather data from staff working in a range of clinic roles, as compared to taking a sampling approach.

The survey included informed consent language that explained the survey to respondents and specified that their participation was voluntary, that they could skip items or stop taking the survey at any time, that personal data would be kept confidential, and that data were for research use only and would not be shared with outside parties. Those invited to take the survey were entered into a sweepstakes for the chance to win one of 13 $100 gift cards for at least one winner per clinic.

To pre-test the survey, some CLARO team members and representatives from the healthcare systems were invited to test the survey and provide feedback about question wording and the length of time it took to complete the survey.

### Recruitment procedures and survey administration

A total of 541 staff at the 13 clinics were sent emails asking for their voluntary survey participation. Inclusion criteria included all staff members including clinicians and other clinic staff members who were likely to interact with patients that may qualify for CLARO. Exclusion criteria were staff who worked in dental, facilities, or other areas in which interaction with potential CLARO patients would be unlikely. The healthcare system leaders most directly involved with the CLARO research team also did not take the survey because of their close involvement with the CLARO RCT; in some cases, they were asked to provide input on the survey prior to launch (e.g., to test the length of time the survey would take). Types of staff that took the survey included medical providers (including physicians, physician assistants, and nurses), behavioral health providers, and other non-clinician staff (including medical assistants and key administrators). This approach allowed for obtaining a relatively comprehensive view of each clinic.

For the three healthcare systems, CLARO RCT start dates (meaning the date when patient enrollment into CLARO was scheduled to begin) ranged from November 2020 to February 2021. The pre-implementation survey was administered to clinic staff members in the 3–4 weeks prior to the date on which RCT enrollment began at each clinic. Because of restrictions related to COVID, no in-person activities in the clinics were possible, and so all facets of survey administration were conducted virtually.

In each system, a clinic champion or administrator sent an email to all staff the week prior to survey launch to let staff know they may receive a legitimate (i.e., not spam) survey invitation email connected to CLARO. These emails also included language that mirrored the IRB-approved survey language to emphasize the voluntary nature of the survey. Survey respondents received an email with a unique URL for the survey from the RAND Survey Research Group. Any respondents who had not yet completed the survey received a weekly email reminder while the survey remained open.

### Response rates

Among the three healthcare systems, response rates ranged from 50 to 68% across systems and 56 to 85% across role (Table 1), which is considered acceptable for a voluntary, organizational survey.^24,25^ Table 7 in Appendix 1 provides demographic information about the respondents.

System 1, a Federally Qualified Health Center (FQHC), contained clinics located in both urban and rural parts of the state. System 2 was also an FQHC with clinics located in a rural part of the state, different than system 1. In system 3, which was affiliated with a university, clinics were located in an urban area.

### Measures

This study adapted previously validated CFIR scales from Fernandez et al.^20^ and Kegler et al.^26^ and incorporated the Brief Opioid Stigma Scale from Yang et al. (Table 2).^27^ At least one scale related to each of the five CFIR domains. The scales from Kegler et al.^26^ were developed and validated by those authors to help researchers increase the likelihood of implementation success during any stage of implementation including during the planning phases. Their set of measures addressed each of the five CFIR domains and were easily adaptable. Similar to Kegler et al.,^26^ Fernandez et al.^20^ developed measures focused on the Inner Setting CFIR domain. Their study provided scales useful for examining facets of culture, climate, leadership, and resourcing. Yang et al.’s^27^ Brief Opioid Stigma Scale, a 6-item scale focusing on perceptions about individual and community-level stigma, was also included in the survey. Though not part of the CFIR, given the focus of the CLARO RCT, the individual section of the stigma scale contributes to the understanding of Inner Setting, and the community-level section, to Outer Setting. These measures provided coverage of concepts across the major CFIR domains. Additionally, the authors reviewed concepts to make sure they made sense to the context of the CLARO RCT. For example, the survey did not include items on constructs such as Design and Quality of Packaging (a CFIR Intervention Characteristic construct) or External Policies and Incentives (a CFIR Outer Setting construct) because these were less salient to the CLARO RCT and to the aim of this research focused on understanding the clinic setting.

All scales used in the survey were 5-point Likert scales (strongly disagree to strongly agree). To adapt these scales to the context of CLARO, minor edits were made to item wording (e.g., using the term “clinic”). Additionally, given the pre-implementation timing of the survey, the constructs chosen reflected the types of antecedent constructs that Damschroder et al.^22^ later identified; for example, items that were evaluative or retrospective were not included (e.g., items like, the intervention was “used by colleagues who were happy with it”^26(p.4184)^) since this survey was administered prior to the start of the intervention.

### Survey analysis

In the survey, all respondents were asked to complete CFIR scale and demographic items. Behavioral health providers (BHPs; include those with LADAC, LCSW, LMHC, LMSW, LPCC, or PsyD credentials) and medical providers (MPs; include those with MD, PA, NP, or RN/LVN credentials) were asked some additional questions at the end of the survey about their clinic workflow (e.g., extent to which their appointments were in-person or virtual). Any respondent that answered 80% or more of the core questions were included in the analysis. This 80% cutoff rule was used because when missing data were analyzed, an 80% cutoff typically included respondents who had answered at least some items in the main sections of the survey (e.g., respondents that answered the CFIR questions but then did not complete the demographic items located at the end of the survey).

In the survey, questions asked about the respondents’ clinic environment, not about the healthcare system as a whole. Responses were aggregated to the healthcare system level to protect the confidentiality of respondents (e.g., some clinics had fewer than 30 staff total), and to facilitate a comparison across the three distinct systems. Furthermore, each system relied on system-wide top leadership teams, and in some instances, staff members worked at more than one site in a system.

Constructs were analyzed based on percent of favorable responses in which ratings of agree or strongly agree were considered favorable in order to gauge relatively strong versus weak context areas. Most survey items were phrased in an affirming way (e.g., “This clinic does a good job…”). Therefore, in most instances, construct scores that fell below 50% favorability (e.g., in which less than 50% agreed or strongly agreed) were considered areas for improvement. Three constructs, complexity and the two stigma scores, were exceptions in that due to question wording, high percent favorable ratings indicated deficits or areas for improvement. Scores were not reversed for those three constructs because it was simpler and clearer to keep those items as is; for example, it was unclear if reverse scoring the items would reflect respondents’ survey selections (e.g., disagreeing or strongly disagreeing that “most people believe that a person who is addicted to opioids cannot be trusted” may not be the same as agreeing or strongly agreeing that “most people believe that a person who is addicted to opioids can be trusted”).

## Results

The main research question addressed how organizational factors varied among the three systems and across staff. Table 3 compares scores across the three healthcare systems. To depict how constructs varied by role (Table 4), respondents from all healthcare systems were combined and then data were broken out by role. This allowed us to examine role-based differences without unintentionally identifying individuals due to small sample sizes.

There were several similarities across the three healthcare systems. Notably, most areas for improvement focused on inner setting (organization level) CFIR constructs. Culture stress, level of resources, and complexity were low-scoring areas when data were analyzed by healthcare system, indicating that these were particularly salient areas for potential improvement in all three systems. Perceived community stigma was consistently high among the three systems, whereas perceived individual stigma was consistently low.

Among the three systems, individual-level factors tended to be areas of strength. For example, openness to innovation (healthcare system average: 92%; role average: 81%) and knowledge/beliefs about the intervention (healthcare system average: 93%; role average: 79%) both had high favorability ratings.

In terms of variation across systems, system 2 results indicated some additional low-scoring areas for improvement in learning climate, leadership engagement, and reflecting and evaluating. Additionally, for some areas for improvement common to the three systems, system 2 had lower favorability scores than the other two systems, such as with level of resources (28% favorable versus 49% for system 1 and 44% for system 3).

As Table 4 indicates, role-based areas for improvement were similar to the system-level comparison data. Culture stress, level of resources, and complexity were low-scoring areas among all roles. BHPs and RNs both indicated relative advantage as a low-scoring construct, and BHPs specifically indicated engaging champions as a low-scoring area to watch. Similar to system-level results, perceived community stigma was consistently high (close to 50%) among those in different roles (with the exception of RN staff members who had an item *n* = 0). Tables 5 and 6 summarize the most salient areas for improvement by system and by role.

## Discussion

This study presents a unique contribution to implementation literature by applying quantitative CFIR measures to assess pre-implementation contextual data about three healthcare systems. Rather than focusing on a single aspect of the CFIR, the survey built on previous research to capture insights from each CFIR domain and added additional insights about stigma perceptions. Surveying staff in different roles allowed for a comprehensive view of clinic context, and the research team used the findings to inform the implementation.

By understanding both weaknesses and strengths, these results provided useful feedback to the CLARO research team about the healthcare systems in the pre-implementation period. Overall, there were several consistencies across the three systems and across roles. Most areas for improvement at the system- and role-levels focused on inner setting (organizational focused) constructs whereas individual-level (staff focused) constructs tended to be areas of strength. Perceived community stigma also tended to score relatively high across systems and across roles. Some survey findings seem analogous to contextual factors identified in previous research such as concerns about resources.^16^

With regard to variation, system 2 results indicated some additional areas for improvement with inner setting and process constructs when compared to systems 1 and 3. Notably, as the CLARO RCT progressed, some challenges with system 2 gradually developed, shedding light on the pre-implementation survey findings. Emergent challenges included a complex and hierarchical leadership approach, combined with high staff burnout and patient skepticism about the health system and providers. As an example, in system 2, the research team eventually learned that while the population included many who could technically qualify for CLARO, many clinic staff believed they would not be able to recruit patients due to perceived stigma in the rural community (e.g., it was highly likely that a given patient would know everyone working in the clinic, from the front desk staff administrator to the pharmacist, so some worried patients may fear a damaged reputation). Implementation was later discontinued in system 2 due to low levels of adoption and fidelity to the intervention model.

There was a fair amount of consistency when comparing survey data by role in that some inner setting and intervention constructs stood out across most roles similar to system-level results. In terms of variation, BHP results indicated that the process construct, engaging champions, was an area for further examination. Additionally, MPs seemed to have more favorable views about relative advantage as compared to BHPs and RNs. The possible reasons for those differences are less clear and would benefit from additional research.

This research builds on the work of Fernandez et al.^20^ and Weir et al.^21^ by not only capturing contextual data from sites participating in an EBP in a pre-implementation stage, but also in the work the research team did to report and discuss the findings with the participating healthcare system leadership. A 2-page summary report was developed for each healthcare system that showed the range of responses for each construct. The goal was to provide the healthcare systems with objective feedback that they could use to reflect on CLARO RCT implementation as well as about their healthcare system writ large. Targeted discussions were held with clinic leaders where reports were discussed.

Discussions with clinic leaders focused on the many apparent strengths as well as areas for improvement including the topics, culture stress, resources, complexity, and perceived community stigma. Strengths were considered areas that clinic leaders and staff members could build on to support the CLARO RCT. For example, while many leaders might worry that employees will be resistant to something new, the high-scoring individual-level characteristic, openness to innovation, showed that staff members in these healthcare systems tended to be open to new initiatives. This finding provided a strong starting point for conversations with staff about CLARO as a new initiative.

In a second example, in one healthcare system, discussions addressed how the survey findings related to ongoing concerns about employee burnout, and what that meant for implementation. CLARO team members met with clinic leaders to talk through the steps that clinic leaders planned to take to support their clinic staff members with issues related to stress, lack of resources, burnout, and related issues like leadership turnover.

Additionally, because there were concerns about community-level stigma in all three healthcare systems, researchers spoke with leaders from each system about the nuances of what community stigma looked like in their geographic areas to help determine the biggest potential challenges related to implementation (e.g., if stigma was prominent among certain types of patients specifically, such as the unhoused).

Through these types of conversations, CLARO team members were able to probe some of the nuances evidenced by survey data to support strategies for the implementation. The CLARO team did not change IRB-approved implementation protocols, but they were able to provide suggestions or ideas about aspects of the implementation that fell within the RCT’s control to some extent (e.g., ideas for champions and other coordinators), and suggestions for clinic leaders in support of their healthcare systems. Survey data discussions were also helpful in building relationships among the CLARO team and clinic leaders.

### Limitations

It is notable that this survey was launched during the period in which COVID cases were rising, prior to availability of vaccines. The participating clinics shifted to primarily virtual visits during the survey period; for some providers, like BHPs, all visits were temporarily virtual. The clinics started to increase in-person health care visits in spring of 2021, shortly after the surveys were closed. This also meant that the researchers could not engage in any in-person activities to inform staff about the survey.

This study faced some additional limitations. First, by attempting to capture pre-implementation data, some responses to items about the intervention were speculative by design. The true extent of barriers (or the emergence of unanticipated barriers) was unknowable at the time. Future research could compare pre-implementation data with follow-up data to track barriers over time. Second, methodological limitations include the potential for response bias (e.g., those with the strongest opinions tend to respond) and missing data (with a voluntary survey, it is not realistic to expect high response rates). Additionally, while surveys allow for breadth of data, they do not allow for depth. Knowing this tradeoff, the authors chose to administer a survey in order to gather a breadth of data across members of the three healthcare systems. Alternative approaches, such as interviews, would have made it more difficult to gather data that reflected a wide range of clinic staff roles.

Third, while there were some functional differences among clinics systems (for example, one system uses a group format for delivering medications for opioid use disorder while another has more limited capacity for in-house counseling), clinics within systems shared many similarities, which could have contributed to the broad similarities found in survey results. Future research can use mixed method techniques to supplement survey data with interview, or other, data to examine a broader range of research questions. Fourth, CFIR’s menu of constructs is valuable in that it can be tailored to a study’s situation; however, at the same time, it would have been too burdensome to test all 37 CFIR constructs in survey form. That meant some constructs that were less relevant to the context of the CLARO RCT had to be omitted. Finally, analysis was aggregated to the healthcare system level; further research could explore ways of taking a deeper look at individual clinic-level differences while still protecting the confidentiality of staff members.

In future studies, researchers can further develop procedures or tools for using pre-implementation CFIR data to inform (1) rapid feedback loops with clinic leadership and (2) implementation strategies. Pre-implementation data can be examined for associations with patient outcomes at a later point in time and can be useful for assessing the generalizability of study findings and planning for future dissemination of collaborative care efforts. Looking at the CFIR data post-implementation can also help to identify features of clinic context that are associated with implementation outcomes; to guide exploration in determining which factors influenced effectiveness outcomes by using the CFIR constructs; and to identify the features of the intervention that were implemented with the greatest success or that were likely to be sustained beyond the RCT.

## Implications for Behavioral Health

Findings from this study have implications for understanding the implementation of complex behavioral health interventions into the primary care context. By using a pre-implementation approach and incorporating stigma measures, the authors contribute to Damschroder et al.’s^22^ goal for the CFIR to evolve over time. This work built on previous literature by using validated scales to increase the understanding of the healthcare systems, and the authors sought insights from staff members in different roles including clinicians and non-clinicians, as compared to solely clinic leaders or just one type of personnel. Past research has shown some CFIR determinants to be correlated with implementation outcomes.^28^ This approach, using an organizational survey to examine healthcare systems, also reflects a practical methodical approach that other multisite studies can use. Additionally, holding constructive discussions with clinic members about survey findings reflects an approach for providing survey feedback to participating clinics and for connecting with clinic representatives on both strengths and potential weakness or challenge areas that could impact the implementation of the RCT. This type of constructive feedback loop can be a helpful takeaway for both researchers and clinicians.

## Figures and Tables

**Table 1:** Response rates (RR) by role, across healthcare system

System	Sample *N*	BHP RR	Medical provider RR	Other staff RR	Total RR
System 1	248	11 (100%)	37 (63%)	77 (43%)	125 (50%)
System 2	117	4 (83%)	17 (63%)	57 (47%)	78 (67%)
System 3	176	6 (70%)	59 (62%)	55 (78%)	120 (68%)
**Total**	**541**	**21 85%)**	**113 (62%)**	**189 (56%)**	**323 (60%)**

BHP stands for behavioral health providers (includes those with LADAC, LCSW, LMHC, LMSW, LPCC, or PsyD credentials), medical providers (includes those with MD, PA, NP, or RN/LVN credentials), and other staff includes other types of staff such as administrators

**Table 2:** Summary of survey measures

Domain	Construct	Brief description*
CFIR: outer setting	Patient needs and resources^1^	Clinics understand patient barriers and facilitators, and prioritize patient needs
CFIR: inner setting	Culture^2^	Clinic culture helps support new initiatives
	Culture stress^2^	Individuals have balanced workloads, low levels of stress
	Learning climate^2^	Clinic engages in organizational learning
	Leadership engagement^2^	Leadership support clinic environment and change efforts
	Resources^2^	Availability of financial resources, training, and staffing
CFIR: intervention characteristics	Relative advantage^1^	Intervention will be more effective than usual care
	Complexity^1^	Intervention WILL require lots of clinic changes *(lower scores are considered favorable for this measure)*
	Compatibility^1^	Intervention will fit into workflow
CFIR: process	Engaging champions^1^	Clinic has active intervention champions and management support
	Goals and feedback^1^	Leaders are engaged with intervention; employees are held accountable
	Reflecting and evaluating^1^	Good communication and data are used support clinic initiatives
	Executing^1^	New initiatives are aligned with clinic mission
CFIR: characteristics of individuals	Knowledge/beliefs about the intervention^1^	Respondent’s view that the intervention makes sense
	Openness to innovation^1^	Respondent’s willingness to try a new approach to care
Stigma	Perceived community stigma^3^ (relates to CFIR outer setting)	Extent to which respondents think “most people in the community” hold stereotypical views *(lower scores are considered favorable for this measure)*
	Perceived individual stigma^3^ (relates to CFIR characteristics of individuals)	Extent to which respondents hold stereotypical views about individuals addicted to opioids *(lower scores are considered favorable for this measure)*

1 Kegler et al. ^26^

2 Fernandez et al. ^20^

3 Yang et al. ^27^

* These definitions are summarized for brevity (see Kegler et al. ^28^ and https://cfirguide.org/constructs/ for longer, more comprehensive definitions)

**Table 3:** Percent of favorable responses by healthcare system

CFIR category	Construct	System 1 favorable (%)	System 2 favorable (%)	System 3 favorable (%)	System 1 *N*	System 2 *N*	System 3 *N*
Outer setting	Patient needs and resources	93	80	74	124	66	120
Inner setting	Culture	73	60	58	125	67	120
	Culture stress	33*	31*	38*	125	65	120
	Learning climate	77	49*	71	125	67	120
	Leadership engagement	70	49*	75	125	67	120
	Level of resources	49*	28*	44*	123	67	120
Intervention characteristics	Relative advantage	60	62	80	63	34	54
	Complexity *(lower scores are considered favorable)*	30	44	53*	63	34	51
	Compatibility	71	71	83	63	34	52
Process	Engaging champions	60	51	72	63	33	53
	Goals and feedback	79	76	85	61	34	52
	Reflecting and evaluating	70	48*	70	125	67	120
	Executing	80	69	79	123	67	119
Individuals’ characteristics	Knowledge/beliefs about the intervention	82	79	83	66	34	54
	Openness to innovation	93	85	99	124	67	119
Perceptions about stigma and access to care *(lower scores are considered favorable)*	Perceived community stigma (relates to outer setting)	42	51*	48	65	34	54
	Perceived individual stigma (relates to inner setting)	4	9	3	65	34	54

* Indicates a score signifying an area for improvement or discussion

**Table 4:** Percent of favorable responses by role

CFIR category	Construct	Total BHP (%)	Total MP (%)	Total RN (%)	Total other (%)	BHP N	MP N	RN N	Other N
Outer setting	Patient needs and resources	90	73	85	87	21	92	20	177
Inner setting	Culture	52	66	55	66	21	93	20	178
	Culture stress	24*	32*	40*	36*	21	93	20	176
	Learning climate	71	72	60	67	21	93	20	178
	Leadership engagement	62	71	70	66	21	93	20	178
	Level of resources	43*	29*	50*	49*	21	93	20	176
Intervention characteristics	Relative advantage	48*	75	40*	72	21	52	10	68
	Complexity *(lower scores are considered favorable for this measure)*	33*	30*	44*	52	21	53	9	65
	Compatibility	52	77	90	78	21	53	10	65
Process	Engaging champions	43*	67	78	63	21	52	9	67
	Goals and feedback	84	82	89	77	19	50	9	69
	Reflecting and evaluating	67	68	55	65	21	93	20	178
	Executing	76	81	75	75	21	93	20	175
Individuals’ characteristics	Knowledge/beliefs about the intervention	67	81	80	87	21	53	10	70
	Openness to innovation	86	97	95	93	21	93	20	176
Perceptions about stigma and access to care *(lower scores are considered favorable for these measures)*	Perceived community stigma (relates to outer setting)	48	46	0	46	21	63	0	69
	Perceived individual stigma (relates to inner setting)	0	2	0	8	21	63	0	69

* Indicates a score signifying an area for improvement or discussion

**Table 5:** Summary of areas for improvement represented by range with a low of less than 50% favorable across the 3 healthcare systems

CFIR category	Construct	% favorable range^a^	*N*
Inner setting	Culture stress	31–38%	310
	Learning climate	49–71%	312
	Leadership engagement	49–75%	312
	Level of resources	28–49%	310
Intervention characteristics	Complexity *(lower scores are considered favorable for this measure)*	30–53%	148
Process	Reflecting and evaluating	48–70%	312
Perceptions about stigma and access to care *(lower scores are considered favorable)*	Perceived community stigma	42–51%	153

a Range of scores from the 3 healthcare systems

**Table 6:** Areas for improvement across all clinic staff by role

CFIR category	Construct	BHP % favorable	MP % favorable	RN % favorable	Other staff % favorable	Clinician^a^ *N*	Other *N*
Inner setting	Culture stress	24%	32%	40%	36%	134	176
	Level of resources	43%	29%	50%	49%	134	176
Intervention characteristics	Relative advantage	48%	75%	40%	72%	83	68
	Complexity	33%	30%	44%	52%	83	65
Process	Engaging champions	43%	67%	78%	63%	82	67

a Clinician *N* is combined to include medical provider, BHP, and RN respondents

## Appendix

**Table 7:** Characteristics of respondents

Characteristics (n = 323)	System 1		System 2		System 3		Total	
Gender	N	Percent	N	Percent	N	Percent	N	Percent
Male	15	12%	10	16%	18	15%	43	14%
Female	104	85%	52	81%	94	80%	250	82%
Nonbinary	0	0%	1	2%	3	3%	4	1%
Other	3	2%	1	2%	3	3%	7	2%
Total	122	100%	64	100%	118	100%	304	100%
Race/ethnicity	N	Percent	N	Percent	N	Percent	N	Percent
Hispanic	69	58%	36	56%	65	56%	170	56%
Non-Hispanic White Only	42	35%	24	38%	40	34%	106	35%
Non-Hispanic Black Only	1	1%	0	0%	1	1%	2	1%
Non-Hispanic Asian Only	3	3%	1	2%	8	7%	12	4%
Non-Hispanic Other	4	3%	1	2%	3	3%	8	3%
Two or more	1	1%	2	3%	0	0%	3	1%
Total	120	100%	64	100%	117	100%	301	100%
Age	N	Percent	N	Percent	N	Percent	N	Percent
20-29	19	15%	12	19%	35	30%	66	22%
30-39	38	31%	17	27%	42	36%	97	32%
40-49	34	28%	14	22%	22	19%	70	23%
50-59	20	16%	12	19%	13	11%	45	15%
60-69	11	9%	8	13%	5	4%	24	8%
70 and above	1	1%	1	2%	0	0%	2	1%
Total	123	100%	64	100%	117	100%	304	100%
Education	N	Percent	N	Percent	N	Percent	N	Percent
No high school diploma or equivalent	2	2%	0	0%	0	0%	2	1%
High school diploma or equivalent	12	10%	8	13%	6	5%	26	9%
Some college, but no degree	39	32%	21	33%	19	16%	79	26%
Associate’s degree (AA, AS)	10	8%	10	16%	8	7%	28	9%
Bachelor’s degree (BA, BS)	4	3%	4	6%	19	16%	27	9%
Master’s degree (MA, MS, M. ENG, M. ED, MSW, MBA)	21	17%	7	11%	13	11%	41	13%
Doctoral degree or equivalent (PhD, EDD)	0	0%	2	3%	7	6%	9	3%
Professional degree beyond a Bachelor’s degree (MD, DDS, DVM, LLB, JD)	29	24%	10	16%	38	32%	77	25%
Other	6	5%	2	3%	7	6%	15	5%
Total	123	100%	64	100%	117	100%	304	100%
Role*	N	Percent	N	Percent	N	Percent	N	Percent
MP	37	30%	17	22%	59	49%	113	35%
BHP	11	9%	4	5%	6	5%	21	7%
Other	77	62%	57	73%	55	46%	189	59%
Total	125	100%	78	100%	120	100%	323	100%
Tenure in position	N	Percent	N	Percent	N	Percent	N	Percent
Less than a year	9	8%	22	30%	24	21%	55	18%
1 to 4 years	48	40%	22	30%	51	45%	121	40%
5 to 10 years	37	31%	19	26%	26	23%	82	27%
More than 10 years	25	21%	10	14%	13	11%	48	16%
Total	119	100%	73	100%	114	100%	306	100%

* As noted in the article, BHP stands for behavioral health providers (includes those with LADAC, LCSW, LMHC, LMSW, LPCC, or PsyD credentials), Medical Providers (includes those with MD, PA, NP, or RN/LVN credentials), and Other Staff includes other types of staff such as administrators
